# A star is torn—molecular analysis divides the Mediterranean population of Poli’s stellate barnacle, *Chthamalus stellatus* (Cirripedia, Chtamalidae)

**DOI:** 10.7717/peerj.11826

**Published:** 2021-07-21

**Authors:** Yaron Tikochinski, Sharon Tamir, Noa Simon-Blecher, Uzi Motro, Yair Achituv

**Affiliations:** 1 Faculty of Marine Sciences, Ruppin Academic Center, Mikhmoret, Israel; 2 The Mina & Everard Goodman Faculty of Life Sciences, Bar-Ilan University, Ramat-Gan, Israel; 3 Department of Ecology, Evolution and Behavior, and the Federmann Center for the Study of Rationality, the Hebrew University of Jerusalem, Jerusalem, Israel

**Keywords:** Mediterranean Sea, EF1, NaKA, Poli’s stellate barnacle, Star barnacle, Strait of Gibraltar, Atlantic current, SNP distribution

## Abstract

Poli’s stellate barnacle, *Chthamalus stellatus* Poli, populates the Mediterranean Sea, the North-Eastern Atlantic coasts, and the offshore Eastern Atlantic islands. Previous studies have found apparent genetic differences between the Atlantic and the Mediterranean populations of *C. stellatus*, suggesting possible geological and oceanographic explanations for these differences. We have studied the genetic diversity of 14 populations spanning from the Eastern Atlantic to the Eastern Mediterranean, using two nuclear genes sequences revealing a total of 63 polymorphic sites. Both genotype-based, haplotype-based and the novel SNP distribution population-based methods have found that these populations represent a geographic cline along the west to east localities. The differences in SNP distribution among populations further separates a major western cluster into two smaller clusters, the Eastern Atlantic and the Western Mediterranean. It also separates the major eastern cluster into two smaller clusters, the Mid-Mediterranean and Eastern Mediterranean. We suggested here environmental conditions like surface currents, water salinity and temperature as probable factors that have formed the population structure. We demonstrate that *C. stellatus* is a suitable model organism for studying how geological events and hydrographic conditions shape the fauna in the Mediterranean Sea.

## Introduction

**Figure 1:**
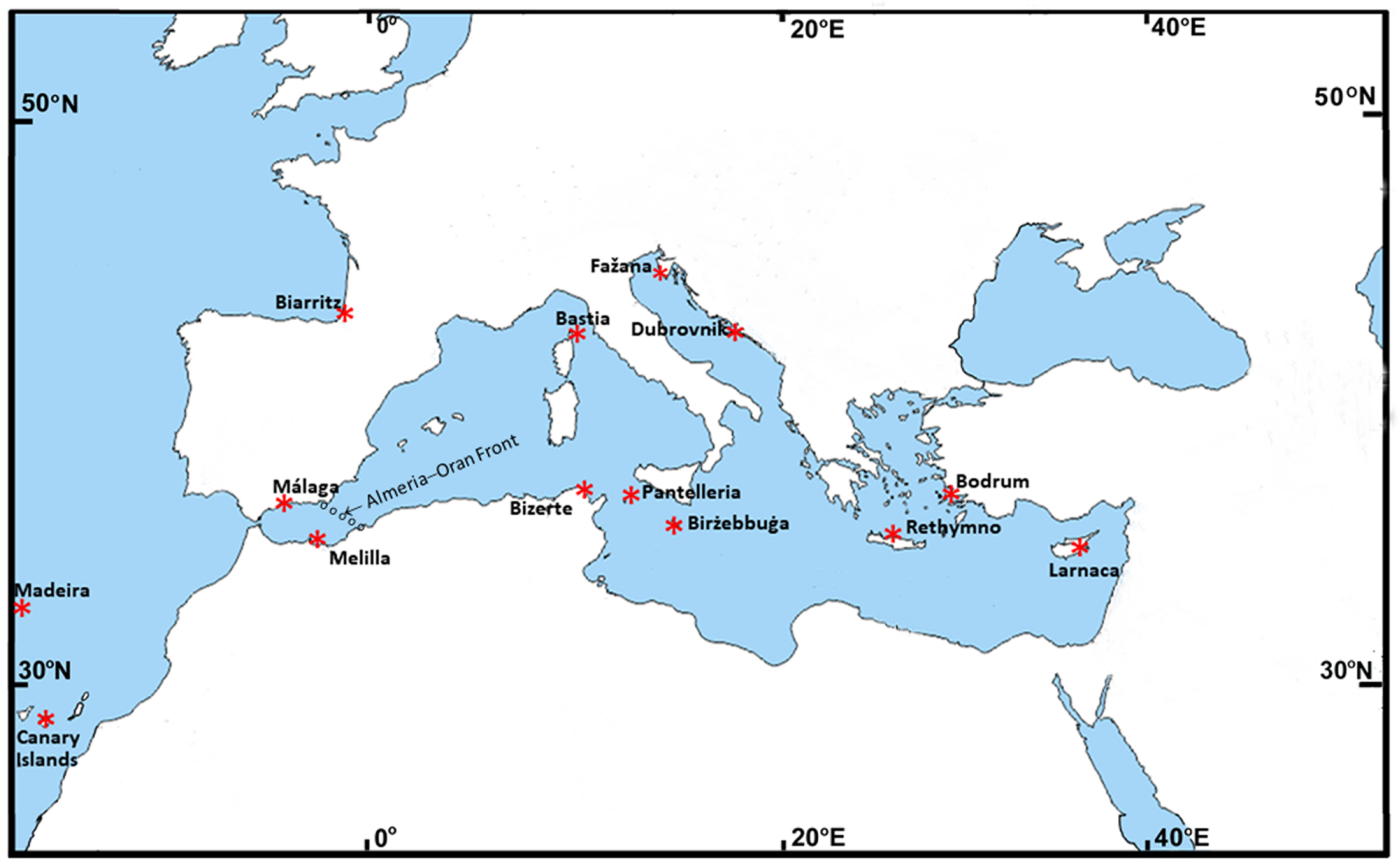
Map of the collection sites. Map showing the 14 collection sites.

Barnacles of the genus *Chthamalus* are a major worldwide component of the rocky intertidal zone of tropical and sub-tropical shores, with few species penetrating into temperate latitudes. Poli’s Stellate barnacle, *Chthamalus stellatus* Poli, is a species with wide geographical range, covering the Mediterranean Sea, the North-Eastern Atlantic coasts, and the offshore Eastern Atlantic islands: Madeira, Azores, and Cape Verde (Southward, 1976; Stubbings, 1967). Recently it was demonstrated that the Cape Verde Islands population is an independent Evolutionary Significant Unit (ESU), a sister clade of *C. stellatus* (Tikochinski et al., 2020). *C. stellatus* is absent from the North-Western Africa Atlantic coast, unlike another species of *Chthamalus*, *C. montagui* Southward, which is also widely distributed in the Mediterranean. Burrows, Hawkins & Southward (1992); Burrows, Hawkins & Southward (1999) suggested that differences in the distribution of these two species and especially the absence of *C. montagui* from the Atlantic islands are related to the lifespan of their pelagic stage. The larvae of *C. stellatus* are larger than those of *C. montagui,* they live longer and can disperse further offshore*.* This strategy appears to allow *C. stellatus* to maintain populations on offshore islands. However, *C. stellatus* could not spread further south to the North-Western African shores and the neighboring islands, presumably because of incompatible salinity and temperature conditions (Bhatnagar & Crisp, 1965). Several molecular studies have aimed to resolve the population structure of Chthamalids in the Mediterranean Sea and the oceanographic forces leading to this structure. Pannacciulli, Bishop & Hawkins (1997) showed that in both *C. montagui* and *C. stellatus* there is a separation between the Atlantic and Mediterranean populations, with *C. montagui* exhibiting a greater separation than that of *C. stellatus*. Since the Mediterranean Chthamalids that are located close to the Strait of Gibraltar resemble the Atlantic ones, they hypothesized that the Almeria–Oran Front in the Alboran Sea (Fig. 1) is a major barrier to larval dispersion and therefore restricts gene flow between the Atlantic and the Mediterranean of the two *Chthamalus* species. The Almeria–Oran Front is regarded as a major transition zone between the Atlantic and the Western Mediterranean for 58 marine species, including *C. montagui* (Patarnello, Volckaert & Castilho, 2007). Based on the mitochondrial COI gene, the population structure of *C*. *montagui in the* North-Eastern Atlantic Ocean, the Mediterranean and the Black Sea (altogether 13 locations) was studied by Pannacciulli et al. (2017). Using three molecular markers, Shemesh et al. (2009) examined the distribution of three Chthamalid species, *C. stellatus*, *C. montagui* and *Euraphia depressa*, in the Eastern Atlantic, the Mediterranean Sea and the Black Sea. Their wide selection of sampling sites revealed a significant genetic structure among the populations of these Chthamalids. However, for *C. stellatus*, the structure was based only on two markers (COI and EF1), each having a small number of sampled individuals (a total of 23 and 40, respectively), which is not sufficient for an adequate population study. In a geographical study of the barnacle *Hexechamaesipho pilsbryi* in Asia, Tsang et al. (2013) have separated the population into two major classes, representing two distinct lineages (the Northern and the Southern lineages), and this separation was based on a single mitochondrial marker, COI. Based on the same single gene, Tsang et al. (2012) recognized in the *Chthamalus malayensis* four genetically differentiated allopatric clades, three of these were already regarded by Tsang et al. (2008) as cryptic species.

Our present study is not supposed to be an evolutionary or a phylogenetic analysis of *C. stellatus*, but rather a description of the genetic structure of the population of this species in the Mediterranean and the adjacent region of the North-Eastern Atlantic. For that purpose, we used two nuclear markers, EF1 and NaKA. While the number of collecting sites (14) is slightly smaller than in the abovementioned studies, they adequately represent both the Eastern and the Western basins of the Mediterranean, as well as a few locations in the Eastern Atlantic. Sample sizes from each location, nevertheless, are quite large, enabling us to perform population genetic analyses and to construct dendrograms which are based on population samples, and not on just a single or a very few individuals from each location. Thus, we can describe and analyze the differences, if they exist, between the various sub-populations, and get a more accurate and more reliable picture of the population structure than that depicted by previous studies, which used only a single or a very few individuals to represent an entire local population.

The genus *Chthamalus* has a wide distribution and intragroup morphological similarity. It has already been used as a model organism for taxonomic, ecological and phylogeographic studies (Pannacciulli, Bishop & Hawkins, 1997; Tsang et al., 2008; Tsang et al., 2012; Shemesh et al., 2009; Wu etal, 2015; Chan et al., 2016). Our present study emphasizes and confirms that *Chthamalus stellatus* is an appropriate model organism for studying how geological events and hydrographic conditions shape the fauna in the Mediterranean Sea.

## Materials and Methods

Samples were collected as previously described in Tikochinski et al. (2020). Specifically, Chthamalus samples were collected in the intertidal rocks by us or were donated by colleagues; the barnacles were fixed and stored in 96% ethanol. The samples used for this study are stored at the Israeli National Natural History Collections at the Hebrew University of Jerusalem (for details see Supplemental Information 1).

We examined *Chthamalus stellatus* populations from 14 different locations (Fig. 1): Five locations are on the Eastern Mediterranean basin—Bodrum (Aegean Sea, Turkey), Dubrovnik (Middle Adriatic Sea, Croatia), Fažana (North Adriatic Sea, Croatia), Larnaca (Cyprus) and Rethymno (Crete). Four locations are on the Western Mediterranean basin—Málaga (North Alboran Sea, Spain), Melilla (South Alboran Sea, Spain), Bizerte (Tunisia) and Bastia (on the southern border of the Ligurian Sea, Corsica). Two locations are in-between the Eastern and the Western basins—Birżebbuġa (Malta) and the island of Pantelleria (Strait of Sicily, Italy). The three remaining locations are in the North-Eastern Atlantic Ocean—Biarritz (Bay of Biscay, France), Canary Islands (Macaronesia) and the island of Madeira (Macaronesia).

Three hundred and forty-eight individuals from these 14 populations were characterized by two nuclear genetic markers—EF1 and NaKA. Each population included at least 15 individuals for each marker (See Table 1). GenBank accession numbers used in our study are MT296012–MT296247 and MT633576–MT633654 for EF1, MT296286–MT296518 and MT633655–MT633737 for NaKA. In addition, we used 38 EF1 sequences and 37 NaKA sequences of the recently described cryptic species of *Chthamalus* from the Cape Verde Islands (Tikochinski et al., 2020) as an outgroup species.

**Table 1:** Number of individuals from each of the 14 populations.

Population	EF1	NaKA
Bastia	21	22
Biarritz	21	25
Birżebbuġa	20	21
Bizerte	23	23
Bodrum	22	25
Canary Islands	25	15
Dubrovnik	24	26
Fažana	27	27
Larnaca	27	28
Madeira	18	15
Málaga	27	26
Melilla	22	23
Pantelleria	21	22
Rethymno	17	18
Total	315	316

### DNA extraction

DNA was extracted from soft tissue of specimens of *Chthamalus stellatus* that were collected from 14 different locations in the Mediterranean and the Eastern Atlantic Sea using the AccuPrep^®^ genomic DNA extraction kit (Bioneer, Daejeon, Korea).

### PCR and sequencing

Two gene segments were amplified using previously described primers for the nuclear genes Na-K-ATPase (NaKA) and elongation factor 1*α* (EF1) (Wares et al., 2009), yielding amplification fragments of 274 and 464bp respectively. Sequencing data were collected as previously described in Tikochinski et al. (2016). Specifically, PCR reactions were carried out in 25-µl reaction volumes containing 1X PCR buffer (including 1.5 mM MgCl_2_), 0.2 mM of each dNTP, 1 µM of each primer, 1 unit of Super-Term Taq polymerase (Hoffmann-La Roche), and about 100 ŋg template DNA. PCR reactions were processed in an MJ Research thermal cycler with the following thermal regime: an initial step of 2 min at 95 °C followed by 35 cycles of 0.5 min at 94 °C, 0.5 min at 57 °C, and 1 min at 72 °C, followed by 3 min at 72 °C and then held at 15 °C. PCR products were visualized on 1.5% agarose gels and sequenced bidirectionally using the PCR primers on an ABI 377 DNA Sequencer (Applied Biosystems, Foster City, CA) following the manufacturer’s instructions.

### Statistical analyses

Forward and reverse sequences obtained for each reaction were aligned. After alignment of all the sequences using BioEdit (Copyright 1986–1993 by Joseph Felsenstein and by the University of Washington), we were left with 425 positions for EF1 and 239 positions for NaKA.

Most genetic studies of barnacles use haploid mitochondrial markers (e.g., COI, the mitochondrial cytochrome c oxidase subunit 1 gene) for inferring relationships among populations. Here we used two nuclear, diploid markers (EF1 and NaKA), and employed three different approaches in our diploid analysis.

The first approach (the ‘genotype-based’ method) was simply to calculate pairwise distances between genotypes using the F84 model of nucleotide substitution (Felsenstein & Churchill, 1996), which differentiates between transitions and transversions and allows for unequal base composition. This was done by choosing the F84 metric in the DNADist©(Ver 3.5c) procedure, which is included in the BioEdit package. Thus, we obtained 49,455 pairwise distances for EF1 and 49,770 for NaKA, and the next step was to compute the means of the 91 between-population distances for each marker. Next, the overall between-population distance matrix was obtained by adding the corresponding distances of each of the two markers using the Euclidean measure (i.e., each entry of the overall matrix was the square root of the sum of squares of the corresponding entries of EF1 and NaKA). Finally, the overall 14 ×14 distance matrix served to construct a population topology tree by furthest neighbor (complete-linkage clustering) amalgamation, using the MVSP software (Version 3.22; Kovach Computation Services, Pentraeth, UK).

The second approach (the ‘haplotype-based’ method) tries to divide each diploid sequence into two haplotypes, using the DnaSP v6 software (Rozas et al., 2017). As a result, each sampled barnacle yielded two distinct haplotypes of the marker. Note, however, that such a division is not necessarily a unique one. The preceding analysis is similar to that commonly used for haploid markers. We used the Molecular Evolution Genetics Analysis (MEGA X 2020) software for obtaining the 198,135 and 199,396 pairwise distances for EF1 and NaKA, respectively, and the next steps follow those described above for the genotype-based method.

The third approach (the ‘population-based SNP-distribution’ method) compares the nucleotide frequency among the various populations, as described in Tikochinski et al. (2020). Specifically, after alignment, we discarded the monomorphic nucleotide positions, and considered only those polymorphic. Thus, we were left with 30 positions for EF1 and 33 positions for NaKA, and the following analysis was done for each marker separately: For each of the 14 populations, we calculated the distribution of the four different nucleotides (A, C, G and T) in each of the nucleotide positions. Next, for each position we compared the distribution of the four different nucleotides between the 14 different populations, by using a distance metric (see below). Thus, we get for each position, 91 pairwise distances. Note that for a pair of populations, the distances at the different positions can be non-independent (due to existing linkage disequilibria between positions). These non-independent distances were averaged over all relevant positions of the marker, to obtain the final pairwise distance between that pair of populations. The results for all pairs of populations were arranged in a 14  × 14 symmetric distance matrix. Again, this was done separately for each marker. The overall between-population distance matrix was obtained by adding the corresponding distances of each of the two markers using the Euclidean measure. This final matrix served for constructing population dendrograms. While the population-based SNP-distribution method is straightforward and relatively easy to perform, it has the disadvantage of breaking existing linkage disequilibria between nucleotides in different positions. Thus, it is not suitable for inferring evolutionary origins and processes. Nevertheless, it can nicely describe the overall existing differences (like differences in SNP distributions) between populations. The approach of combining SNP’s, regardless of possible linkage, for population studies, is used in NGS methods like RAD sequencing (Chen et al., 2020; Longo et al., 2020, and many more). Many population studies use differences in SNP distributions to distinguish between various populations within a species, e.g., in red clovers (Ergon et al., 2019), in marine species (Weist et al., 2019), in humans (Mao et al., 2017) and others.

For the SNP-distribution method we used three different distance measures, the squared Euclidean distance, a modified squared chord distance and the Manhattan (or city block) distance. If *x*_1_, *x*_2_, *x*_3_ and *x*_4_ are the proportions of A, C, G and T in population 1, and *y*_1_, *y*_2_, *y*_3_ and *y*_4_ are these proportions in population 2, then: the squared Euclidean distance = ${\mathop{\sum }\nolimits }_{i=1}^{4}({x}_{i}-{y}_{i})^{2}$; the modified squared chord distance = ${\mathop{\sum }\nolimits }_{i=1}^{4}(\sqrt{{x}_{i}}-\sqrt{{y}_{i}})^{2}$ (which is actually the squared length of the chord connecting $(\sqrt{{x}_{1}},\sqrt{{x}_{2}},\sqrt{{x}_{3}},\sqrt{{x}_{4}})$ and $(\sqrt{{y}_{1}},\sqrt{{y}_{2}},\sqrt{{y}_{3}},\sqrt{{y}_{4}})$ on the unit four-dimensional sphere); and the Manhattan distance = ${\mathop{\sum }\nolimits }_{i=1}^{4} \left\vert {x}_{i}-{y}_{i} \right\vert $. In all cases, we applied the furthest neighbor (complete-linkage clustering) amalgamation, and generated topology trees, using the MVSP software.

For each population we calculated, separately for each marker, the mean number of different alleles per position. We then averaged over the two markers to obtain the overall mean number of different alleles per position in this population. In addition, we calculated for each population the mean expected heterozygosity of a marker, defined as $1- \frac{1}{N} {\mathop{\sum }\nolimits }_{k=1}^{N}{\mathop{\sum }\nolimits }_{i=1}^{4}{x}_{i,k}^{2}$, where *x*_1,*k*_, *x*_2,*k*_, *x*_3,*k*_ and *x*_4,*k*_ are the proportions of A, C, G and T in position *k* (*k* = 1, 2, …, *N*, where *N* is the number of positions in the marker). We then averaged the measures of the two markers, to obtain the expected heterozygosity at the relevant population. Similarly, for each population, we calculated the percentage of polymorphic positions in each marker, and then averaged the percentages of the two markers, to obtain the polymorphism measure of the relevant population. Since distances are non-independent, comparing mean distance within groups to the mean distance among groups was performed by computer simulations (1,000 random permutations in each comparison). Other statistical tests (Kolmogorov–Smirnov and one-way ANOVA) were carried out using IBM SPSS Statistics 26. All *p*-values are given for a two-tailed alternative.

## Results

The various between-population distance matrices are presented in Supplemental Information 2. They are the basis for constructing the population dendrograms.

**Figure 2:**
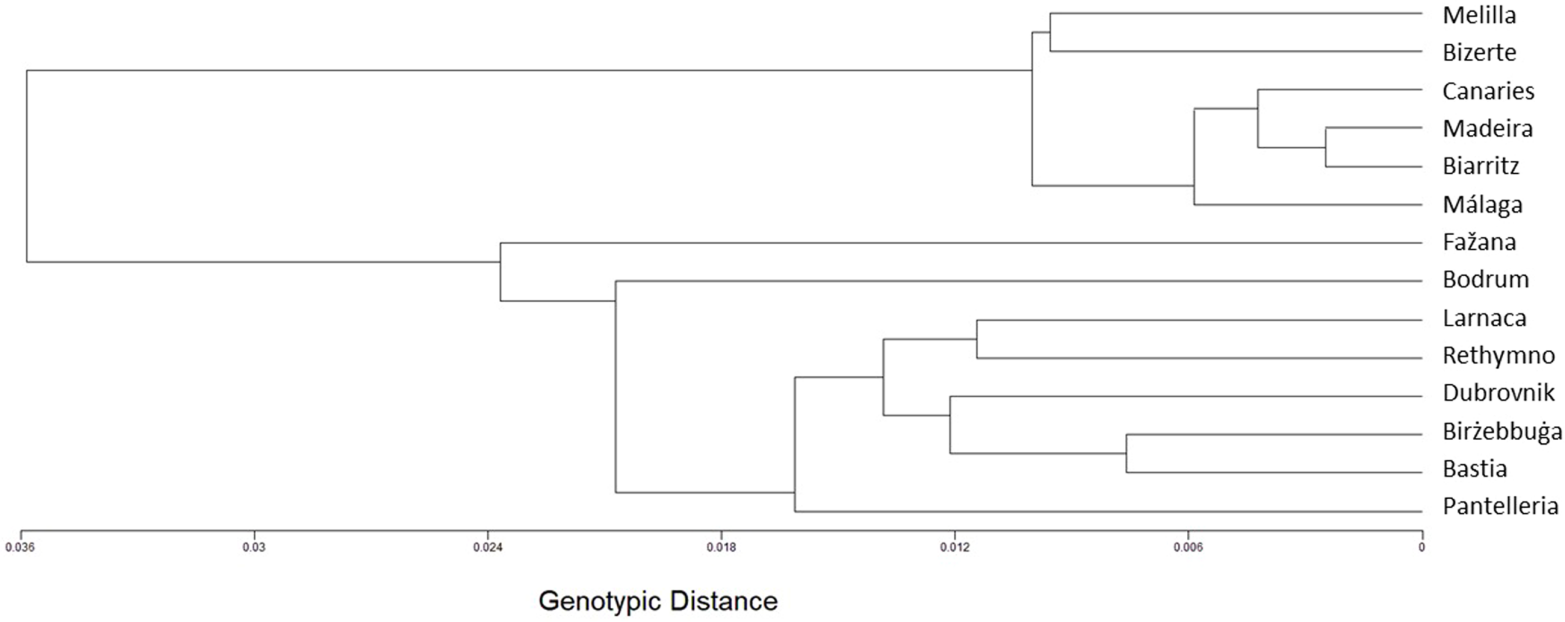
A genotype-based tree of the *Chthamalus stellatus* populations. A topology tree depicting the hierarchical relationship between the 14 *Chthamalus stellatus* populations using the genotype-based distances (see text) and furthest-neighbor clustering.

The genotype-based method displays a geographic structure, dividing the populations between west and east (Fig. 2). The western cluster includes the Eastern Atlantic populations of Biarritz, the Canaries and Madeira, together with the Western Mediterranean populations of Málaga, Melilla and Bizerte. The eastern cluster includes the Eastern Mediterranean populations of Bodrum, Dubrovnik, Fažana, Larnaca and Rethymno, together with Bastia, Birżebbuġa and Pantelleria.

**Figure 3:**
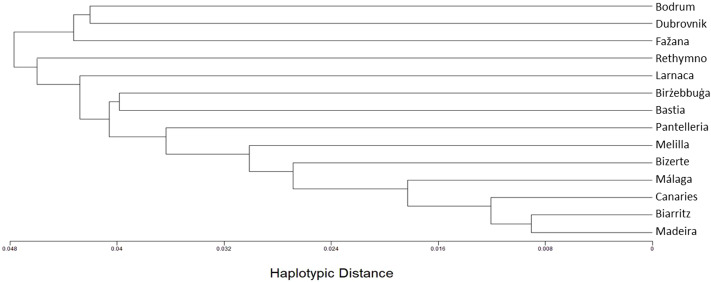
A haplotype-based tree of the *Chthamalus stellatus* populations. A topology tree depicting the hierarchical relationship between the 14 *Chthamalus stellatus* populations using the haplotype-based distances (see text) and furthest-neighbor clustering.

A less distinctive geographic structure is displayed by the haplotype-based method (Fig. 3). Nevertheless, the genotype-based eastern cluster populations (i.e., Biarritz, the Canaries, Madeira, Málaga, Melilla and Bizerte) are clumped together also in this method.

**Figure 4:**
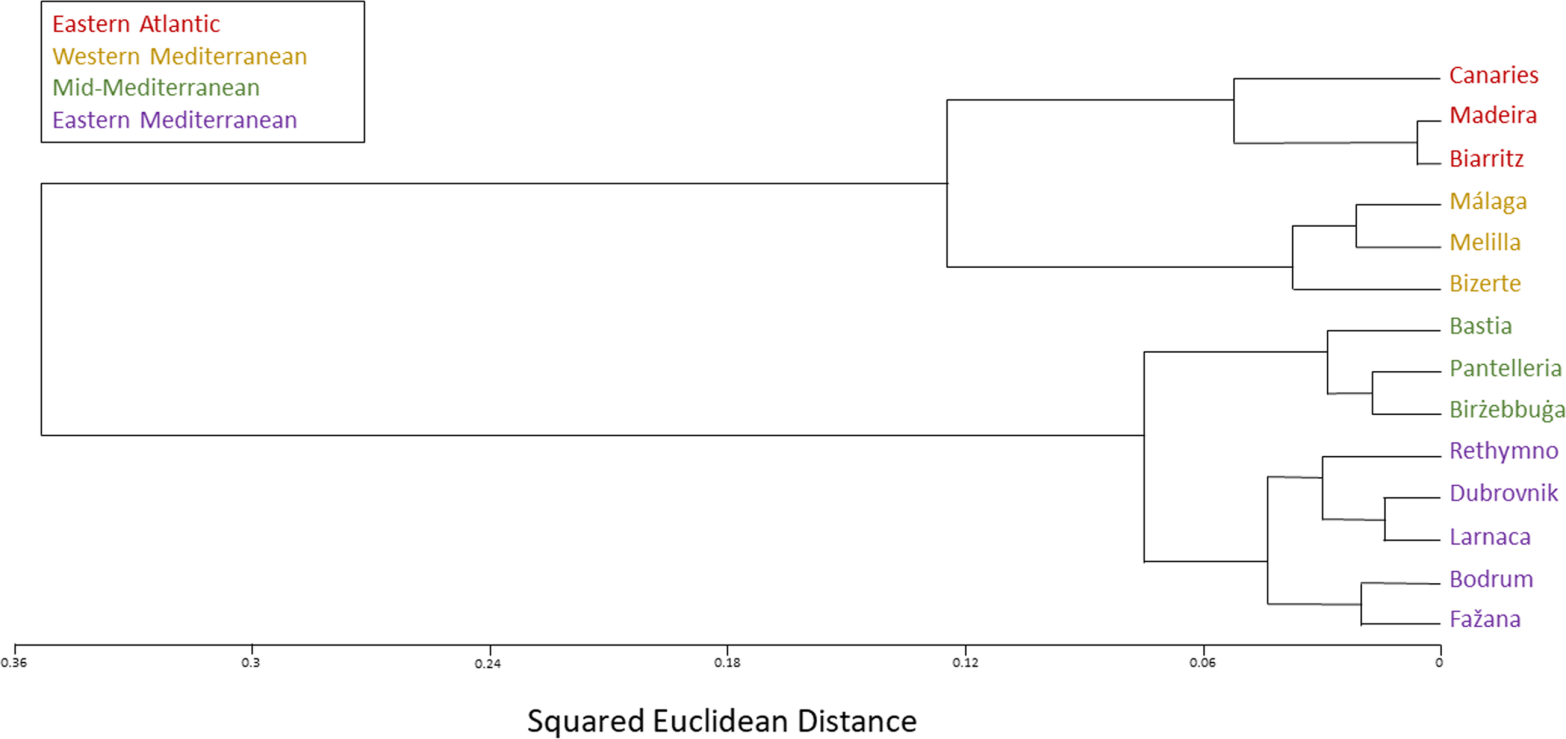
*Chthamalus stellatus* populations SNP population method tree with squared Euclidean distance and furthest neighbor clustering. A topology tree depicting the hierarchical relationship between the 14 *Chthamalus stellatus* populations based on the SNP population method (see text), with squared Euclidean distance and furthest neighbor clustering.

**Figure 5:**
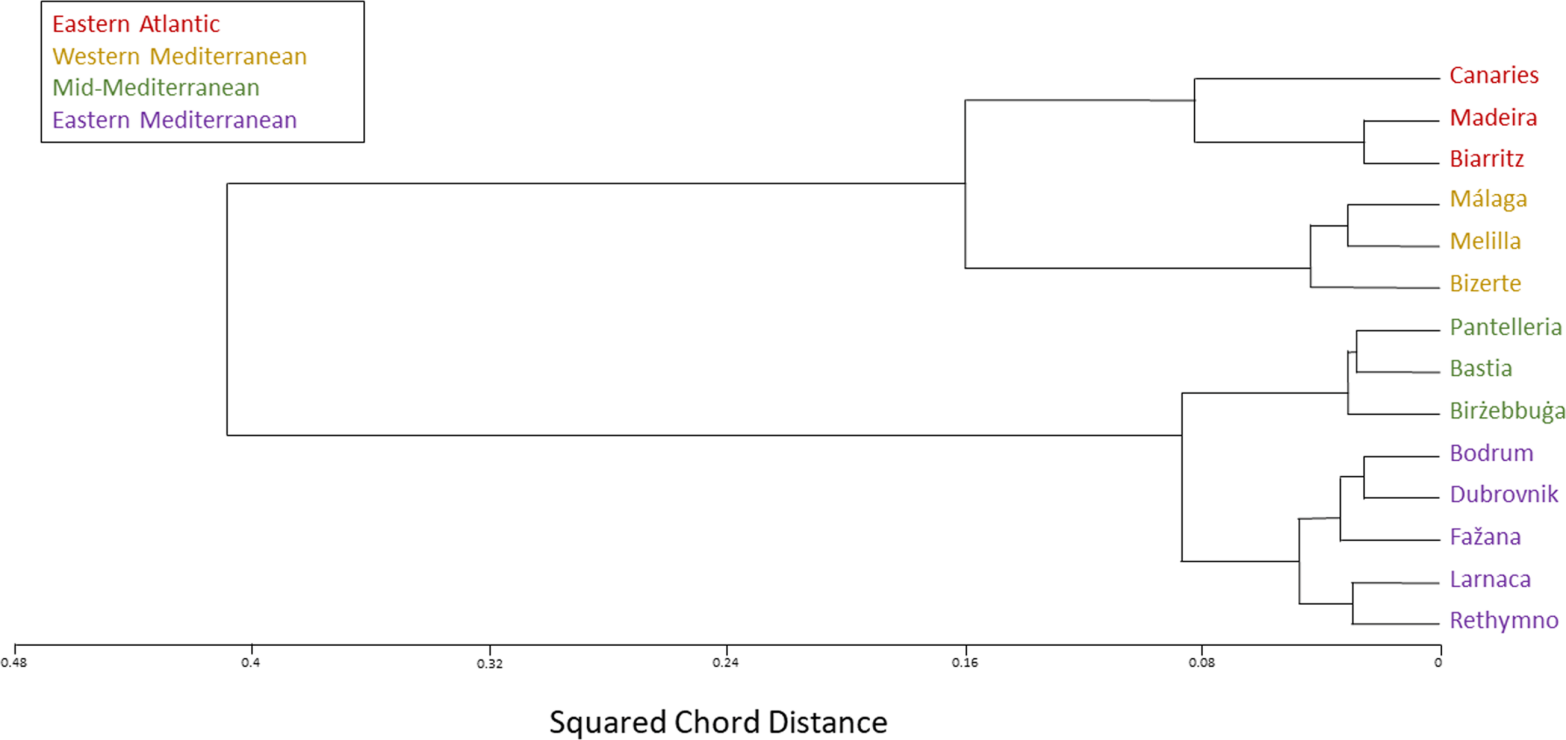
*Chthamalus stellatus* populations SNP population method tree with squared chord distance and furthest neighbor clustering. A topology tree depicting the hierarchical relationship between the 14 *Chthamalus stellatus* populations based on the SNP population method (see text), with squared chord distance and furthest neighbor clustering.

**Figure 6:**
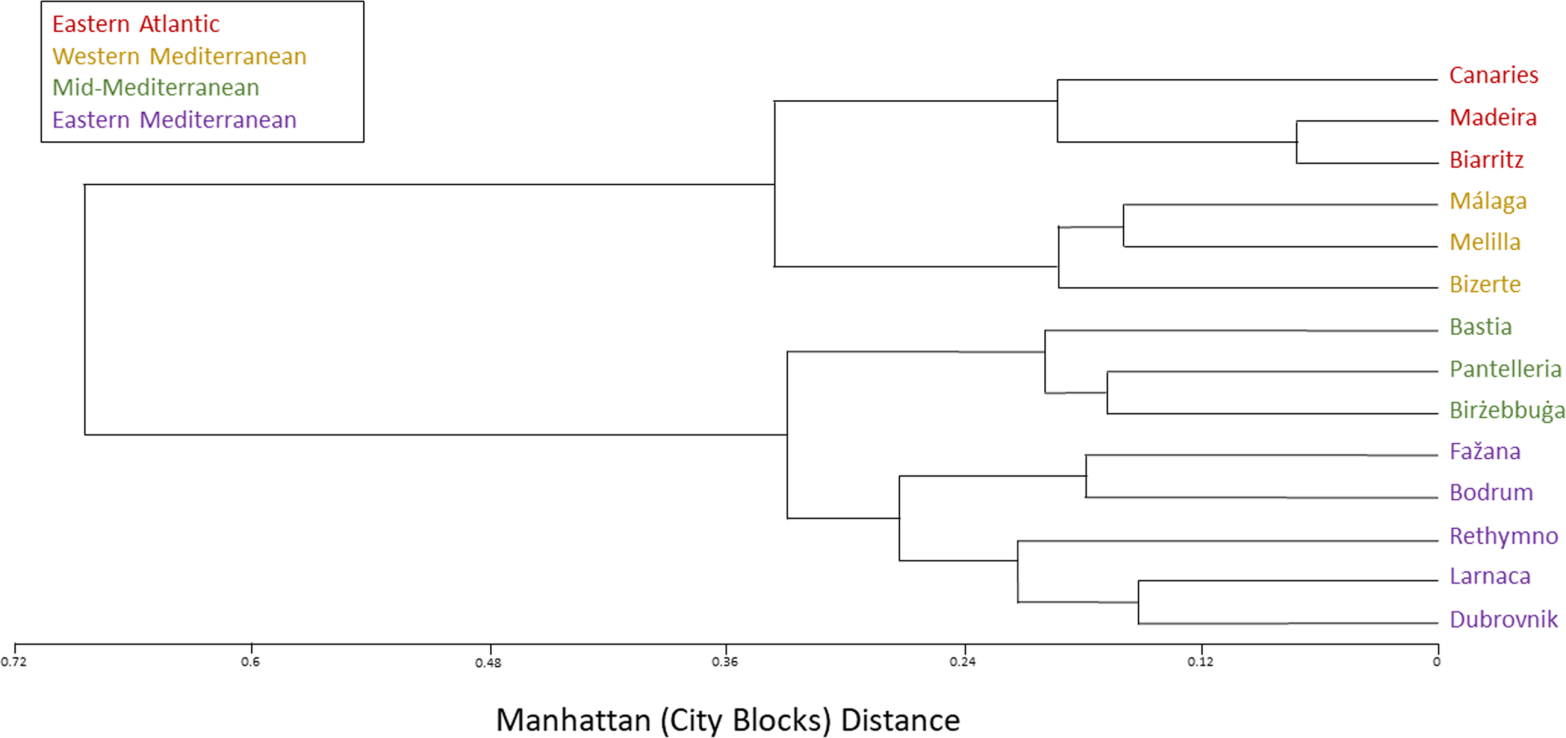
*Chthamalus stellatus* populations SNP population method tree with the Manhattan (city-blocks) distance and furthest neighbor clustering. A topology tree depicting the hierarchical relationship between the 14 *Chthamalus stellatus* populations, based on the SNP population method (see text), with the Manhattan (city-blocks) distance and furthest neighbor clustering.

The SNP population-based method displays a clear-cut division into two major clusters, western and eastern. Moreover, each of these two major clusters is distinctly divided into two smaller clusters: The Eastern Atlantic cluster (Biarritz, the Canaries and Madeira) and the Western Mediterranean cluster (Bizerte, Málaga and Melilla) are the two clusters within the western major cluster, whereas the Mid-Mediterranean cluster (Birżebbuġa, Pantelleria and the Ligurian port of Bastia) and the Eastern Mediterranean cluster (Bodrum, Dubrovnik, Fažana, Larnaca and Rethymno) are the two clusters within the eastern major cluster (Figs. 4–6).

Note that the population-based SNP-distribution method is less sensitive to the inclusion of some rare individuals (that might have migrated or drifted into the population from other distant areas, for example) than the individual-based (genotype or haplotype) methods. To illustrate this, let us focus on a single position in a population of *n* haplotypes. Suppose that in this position, all *n*–1 haplotypes are identical, but one is different. This produces a variation of size $ \frac{1}{n} $ in the SNP-distribution, but twice as large (i.e., $ \frac{(n-1)}{ \frac{1}{2} n(n-1)} = \frac{2}{n} $) in the individual-based pairwise distances. In simple words, the SNP-distribution method gives relatively less weight to rare individuals in a population, and this emphasizes the more common feature of the population.

In accordance with the results of the multi-variate analyses, as presented by the population dendrograms (Figs. 4–6), we divided the 14 populations into four groups: EA (the Eastern Atlantic cluster), WM (the Western Mediterranean cluster), MM (the Mid-Mediterranean cluster) and EM (the Eastern Mediterranean cluster). For each of the three distance matrices–the squared Euclidean, the squared chord and the Manhattan–we computed the mean distance within groups and compared it to the mean distance among groups. Since distances are non-independent, the comparisons were performed by computer simulations (1,000 random permutations in each matrix). For each distance measure, the mean distance among groups was significantly larger than the mean distance within groups (estimated *p* < 0.001 in each case). The results are presented in Table 2.

**Table 2:** SNP population-based method: Mean population distance within clusters compared to mean distance among clusters.

**Mean distance**	**Squared euclidean**	**Squared chord**	**Manhattan city-blocks**
Within EA cluster	0.0185	0.0362	0.0869
Within WM cluster	0.0186	0.0244	0.1186
Within MM cluster	0.0142	0.0202	0.1223
Within EM cluster	0.0184	0.0238	0.1428
Mean within clusters	0.0178	0.0253	0.1269
Mean between clusters	0.0883	0.1053	0.2694
Significance	*p* < 0.001	*p* < 0.001	*p* < 0.001

**Notes.**

EA Eastern Atlantic WM Western Mediterranean MM MidMediterranean EM Eastern Mediterranean

*p*-values were estimated by computer simulations, 1,000 random permutations in each case.

The mean number of different alleles per position, the mean expected heterozygosity measures and the mean percentage of polymorphic positions in each population are presented in Table 3.

**Table 3:** Different alleles per position, expected heterozygosity and polymorphic positions in each population. Mean number of different alleles per position, mean expected heterozygosity and the mean percentage of polymorphic positions in each of the 14 populations.

Population	Number of alleles	Expected heterozygosity	Percent polymorphism
Bastia	1.870	0.198	74.394
Biarritz	1.391	0.072	37.576
Birżebbuġa	1.785	0.215	65.909
Bizerte	1.721	0.151	61.212
Bodrum	1.979	0.229	79.091
Canary Islands	1.389	0.083	35.606
Dubrovnik	1.933	0.228	75.758
Fažana	1.997	0.228	82.273
Larnaca	1.902	0.226	77.576
Madeira	1.302	0.054	28.636
Málaga	1.762	0.144	69.697
Melilla	1.864	0.168	70.758
Pantelleria	1.917	0.203	77.424
Rethymno	1.902	0.241	72.576

**Figure 7:**
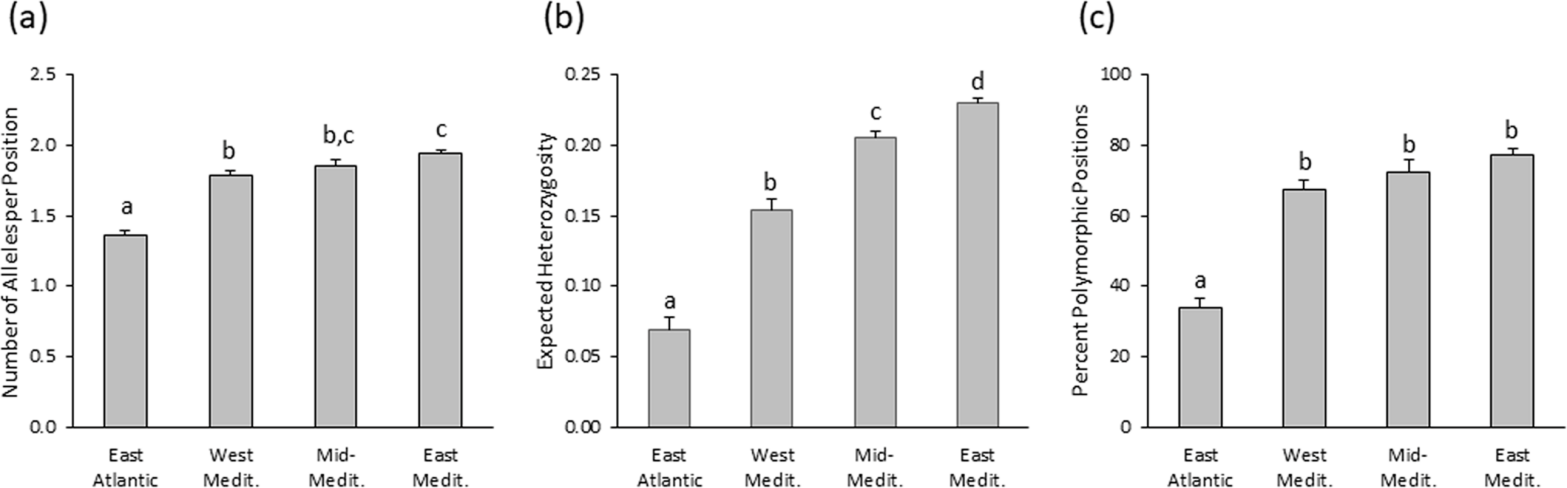
Comparing between the Eastern Atlantic, the Western Mediterranean, the Mid-Mediterranean and the Eastern Mediterranean populations. Comparing between the Eastern Atlantic, the Western Mediterranean, the Mid-Mediterranean and the Eastern Mediterranean populations with respect to (A) number of alleles per position, (B) expected heterozygosity, (C) percent of polymorphic positions. Different letters indicate differences significant at the 5% level (taking account of the Bonferroni correction for multiple comparisons).

The distribution of the number of alleles per position is not statistically different from a normal distribution (Kolmogorov–Smirnov test, *p* = 0.364). The means (± se) of each cluster are: 1.361 ± 0.030, 1.782 ± 0.042, 1.857 ± 0.039 and 1.942 ± 0.020, for EA, WM, MM and EM, respectively (see Fig. 7A). Using a one-way ANOVA for testing the differences in the mean number of alleles per position between these four clusters, we get *F*_3,10_ = 68.849, *p* < 0.001. Post-hoc: EA vs. WM *p* < 0.001; EA vs. MM *p* < 0.001; EA vs. EM *p* < 0.001; WM vs. MM *p* = 0.841; WM vs. EM *p* = 0.020; MM vs. EM *p* = 0.408.

The distribution of the expected heterozygosity is not statistically different from a normal distribution (Kolmogorov–Smirnov test, *p* = 0.464). The means (±  se) of each cluster are: 0.069 ± 0.008, 0.154 ± 0.007, 0.205 ± 0.005 and 0.230 ± 0.003, for EA, WM, MM and EM, respectively (see Fig. 7B). Using a one-way ANOVA for testing the differences in expected heterozygosity between these four clusters, we get *F*_3,10_ = 169.899, *p* < 0.001. Post-hoc: EA vs. WM *p* < 0.001; EA vs. MM *p* < 0.001; EA vs. EM *p* < 0.001; WM vs. MM *p* = 0.001; WM vs. EM *p* < 0.001; MM vs. EM *p* = 0.041

The distribution of the percentage of polymorphic positions is not statistically different from a normal distribution (Kolmogorov–Smirnov test, *p* = 0.295). The means (±  se) of each cluster are: 33.9% ± 2.7%, 67.2% ±3.0%, 72.6% ± 3.4% and 77.5% ± 1.62%, for EA, WM, MM and EM, respectively (see Fig. 7C). Using a one-way ANOVA for testing the differences in the percentage of polymorphic positions between these four clusters, we get *F*_3,10_ = 57.722, *p* < 0.001. Post-hoc: EA vs. WM *p* < 0.001; EA vs. MM *p* < 0.001; EA vs. EM *p* < 0.001; WM vs. MM *p* >0.999; WM vs. EM *p* = 0.084; MM vs. EM *p* >0.999.

## Discussion

Our study presents for the first time a detailed and more established picture of *Chthamalus stellatus* population distribution in the Mediterranean Sea. Previous studies (Crisp, Southward & Southward, 1981; Pannacciulli, Bishop & Hawkins, 1997; Shemesh et al., 2009) used only a single or a very few individuals to represent each local population. Using 63 SNPs in 14 populations, each consisting of 15–28 specimens, enabled us to get a more reliable picture of this barnacle’s populations in the Mediterranean as well as the Eastern Atlantic.

**Figure 8:**
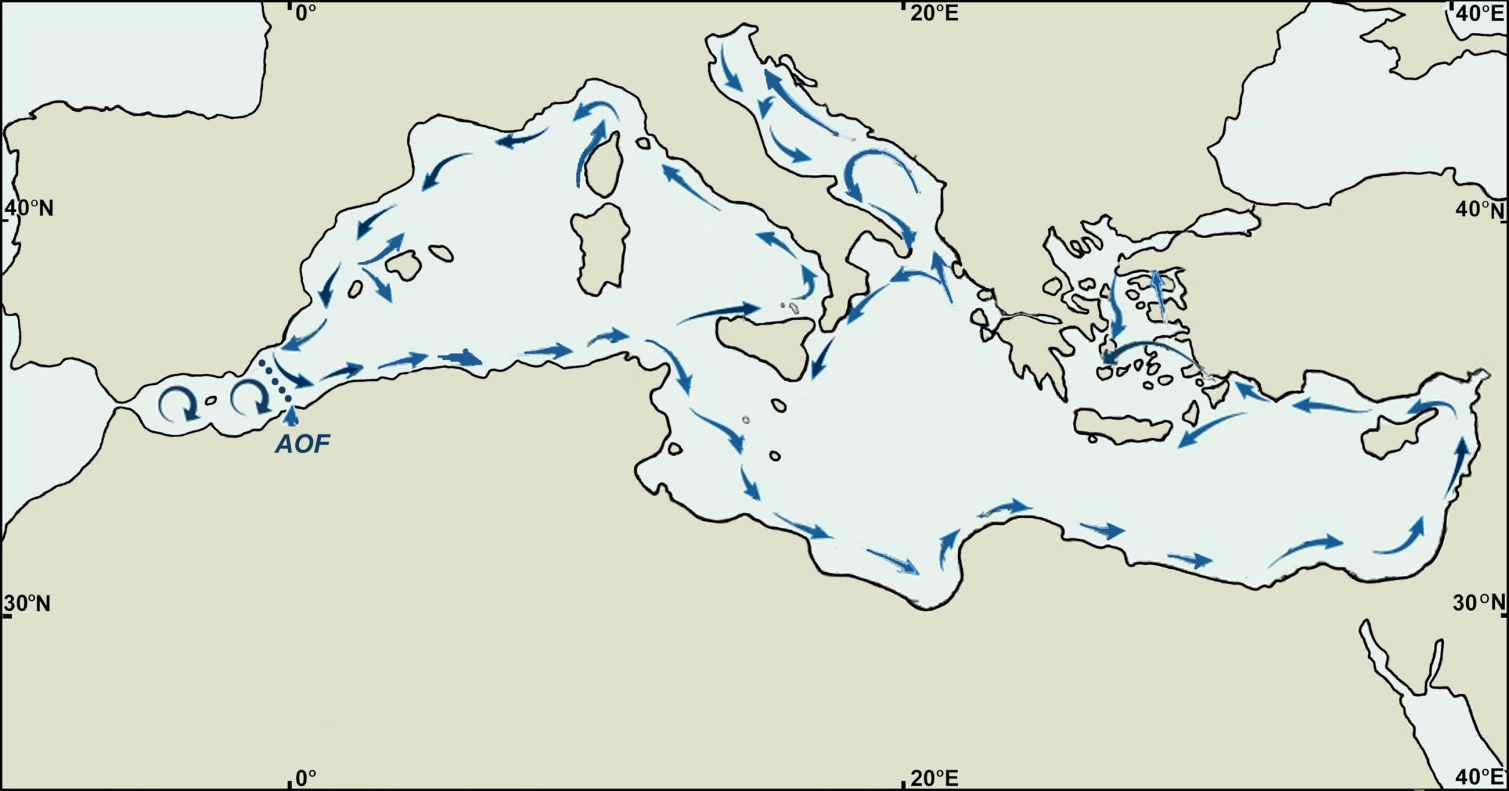
Surface circulation in the Mediterranean. The Almeria-Oran front is shown by the dotted line. [Redrawn from UNEP/MAP (2012), after Millot & Taupier-Letage (2005)].

The various analyses performed in our study display a geographic structure, dividing the populations between west and east. The SNP population-based analysis (Figs. 4–6) further separates the two major western and eastern clusters into four smaller clusters. The Eastern Atlantic (EA) cluster consists of the western peripheral populations of *C. stellatus*, namely, Biarritz, the Canaries and Madeira. This cluster was long-established in previous studies that show the separation between the Atlantic and the Mediterranean populations of *C. stellatus* (Crisp, Southward & Southward, 1981; Pannacciulli, Bishop & Hawkins, 1997; Shemesh et al., 2009). The Western Mediterranean (WM) cluster, which consists of Bizerte, Málaga and Melilla, is a sister cluster to the EA cluster. Two of the cluster’s locations, Málaga and Melilla, are geographically adjacent to the Strait of Gibraltar, and therefore naturally influenced by the surface current entering from the Atlantic and flowing along the Northern Africa coast (UNEP/MAP, 2012) (Fig. 8). The third location (Bizerte) is geographically quite distant, closer to the Mid-Mediterranean locations studied here, but probably still influenced by the flow along the coast. The resemblance between the Atlantic populations and a distant Mediterranean population of barnacles is reported here for the first time. The Eastern Mediterranean (EM) cluster, that consists of Bodrum, Dubrovnik, Fažana, Larnaca and Rethymno, is well-defined in all our SNP population-based analyses. The Mid-Mediterranean (MM) cluster consists of Bastia, Birżebbuġa and Pantelleria. Bastia, at the North-Western coast of Corsica, belongs geographically to the Western Mediterranean basin. The other two MM locations are also close to the Western Mediterranean. Nevertheless, the MM cluster emerged as a sister group to the EM cluster in all our SNP population-based analyses. While this is not surprising for Birżebbuġa and Pantelleria, the Bastia population is an integral part of this cluster. This finding is supported by a previous *C. stellatus* study, clustering Bastia, Genoa and nearby locations with MM populations and not with the Atlantic cluster (Pannacciulli, Bishop & Hawkins, 1997).

**Figure 9:**
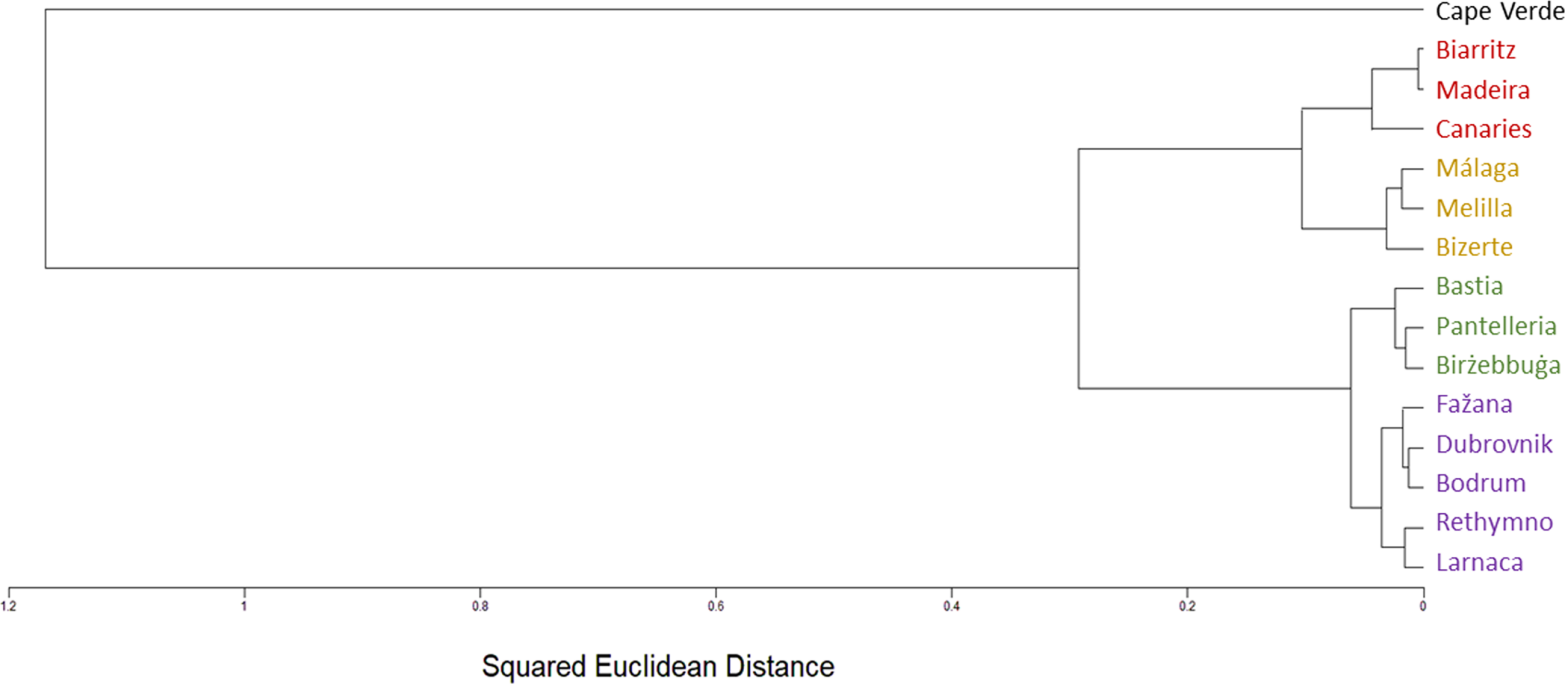
*Chthamalus stellatus* populations and the cryptic species of *Chthamalus* from Cape Verde SNP population method tree with squared Euclidean distance and furthest neighbor clustering. A topology tree depicting the hierarchical relationship between the 14 *Chthamalus stellatus* populations and the cryptic species of *Chthamalus* from Cape Verde (Tikochinski et al., 2020) as an outgroup species. The tree is based on the SNP population method, with squared Euclidean distance and furthest neighbor clustering.

Using the recently described cryptic species of *Chthamalus* from the Cape Verde Islands (Tikochinski et al., 2020) as an outgroup species, gives a relative magnitude to the population structure of *C. stellatus* described in our study (Fig. 9). Indeed, the genetically and geographically based structure can be considered as a fine tuning, within species distribution.

Marine organisms exhibit a variety of dispersal modes (Winston, 2012). However, establishment of new barnacle populations, as well as other sessile animals, mainly occurs by current-assisted larval distribution (Johannesson, 1988). The pelagic stage of *C. stellatus* is about 22 days, allowing for extensive connectivity between populations (Pannacciulli, Manetti & Maltagliati, 2009). The well-documented surface current, coming from the Atlantic Ocean and entering the Mediterranean through the Strait of Gibraltar (Fig. 8) can easily bring barnacle larvae to the shores of Málaga and Melilla. Previous studies have included these locations as part of the Atlantic region population, separated from the rest of the Mediterranean by the Almeria-Oran Front (Pannacciulli, Bishop & Hawkins, 1997). But, according to our results, it appears that the Almeria–Oran Front is not impermeable to the propagules of *C. stellatus* which is reflected in the resemblance of the Western Mediterranean populations of both sides of the front. Patarnello, Volckaert & Castilho (2007) reviewed over 20 population studies of 58 different marine species across the Atlantic-Mediterranean range, aiming to comprehend phylogeographical patterns, including potential barriers in the Mediterranean Sea. The patterns obtained from their data were very diversified, even between closely related species. The three major patterns were (i) full congruence between Atlantic and Mediterranean clades; (ii) distinct Atlantic and Mediterranean clades, where the Almeria–Oran front serves as the Atlantic–Mediterranean phylogeographical break; (iii) an Eastern Mediterranean clade that is distinct from the Western Mediterranean and Atlantic Ocean clade where the Sicily Strait and the Messinian Strait serve as a phylogeographical boundary (Fig. 8). (see also (Villamor, Costantini & Abbiati, 2014)). Our results further expand the second and third models of Patarnello, Volckaert & Castilho (2007) and divide the populations to four clusters. In contrast to the previous above-mentioned analyses, that are based on a limited number of markers and small samples of specimens representing each population, our results that are based on two informative markers and a large population sample, look more reliable.

The Atlantic current, entering through the Strait of Gibraltar, extends east along the shores of Northern Africa (Hamad, Millot & Taupier-Letage, 2006; Millot & Taupier-Letage, 2005; Poulain et al., 2013) (Fig. 8) elucidates the resemblance between Bizerte and the other WM cluster populations and the influence by the sister cluster of the EA populations. This Atlantic current system is likely to assist larval distribution entering the Mediterranean. It appears that the influence of this current does not spread further east. The EM cluster is well defined and spans over a large part of the Mediterranean, from the eastern shores through the Aegean Sea and north into the Adriatic Sea. This part of the Mediterranean is influenced by the Asia Minor current as well as some cyclonic and anti-cyclonic gyres (Pinardi et al., 2006) and we therefore witness increased connectivity between the different populations. The northern part of the Western Mediterranean is influenced by currents from the area of Sicily (Pinardi et al., 2006), and therefore it is not clear why the population resembles the MM populations and not a WM one like Bizerte. The mixing of water by the mesoscale gyres in the Tyrrhenian sea (Fig. 8) may contribute to the resemblance between the Bastia population and that of the other MM populations.

We may also speculate that the separation between the Western and Eastern Mediterranean populations of *C. stellatus* is a result of the geological history of the Mediterranean. One of the most conspicuous events that shaped the composition of flora and fauna of the Mediterranean is the Messinian Salinity Crisis (MSC) that started at the end of the Miocene, about 6 million years ago (Krijgsman et al., 1999) when the connection between the Mediterranean and the Atlantic was cut. The water balance of the Mediterranean was, and still is, deficient and is compensated by the Atlantic inflow through the Strait of Gibraltar. The Messinian Salinity Crisis ended in the Zanclean flood, occurred 5.33 million years ago when the Strait of Gibraltar opened and have refilled the Mediterranean Sea. During the MSC, the water level and salinity of the Mediterranean fluctuated and it was dried up and refilled repeatedly during the few million years of the Messinian stage, leaving behind lakes of different salinities (Hsu, Ryan & Cita, 1973). Some of these lakes might be a refuge, inhabited by resistant organism like intertidal barnacles that naturally withstand fluctuating temperatures and salinities. We may propose that eastern and middle populations of *C. stellatus* are a relic of the MSC lakes while the WM populations resemble the Atlantic “invaders” arriving like many other organisms in the Zanclean flood.

In order to better understand how these populations were shaped, the selective pressures of the habitats, like salinity and temperature, must be considered. When compared to other species of barnacles (Bhatnagar & Crisp, 1965), *C. stellatus* was found to be better adapted to higher temperatures while less inhabiting lower salinity niches. Low salinity has been correlated with reduced number of eggs per brood in *C. stellatus* (Barnes & Barnes, 1965). These adaptive advantages can contribute to a bigger, stable and more polymorphic population in the higher salinity and temperature conditions of the EM. Indeed, the EM populations have more alleles per position and their expected heterozygosity is significantly higher than all other populations (Fig. 7A & 7B). The EA populations, on the other hand, have significantly smaller number of alleles per position, lower expected heterozygosity and lower percent of polymorphic positions (Fig. 7A–7C), presumably reflecting the adaptive difficulties of *C. stellatus* in the lower salinity and temperature of the East Atlantic Ocean. The Atlantic current influence on the Mediterranean is also evident in the low salinity region stretching from the Strait of Gibraltar along the Northern Africa shores to Bizerte (Fig. 8). As expected from this, the WM populations do have less alleles per position and their expected heterozygosity is significantly lower than the EM populations. The environmental conditions may also explain the establishment of EM populations of *C. stellatus* in the western part of the Mediterranean. Mean surface salinity and temperature of the Northern Tyrrhenian and the Ligurian Seas are the highest in the Western Mediterranean Basin (UNEP/MAP, 2012, p 23). These favorable conditions could allow for the establishment of a polymorphic population like the one in Bastia.

Patarnello, Volckaert & Castilho (2007) have concluded that genetic diversity does not necessarily decrease in a direction either from the Atlantic Ocean to the Mediterranean or even to the Adriatic Sea. The higher genetic variability of the EM population can be partially explained by the central-marginal hypothesis (Carson, 1955). The hypothesis claims that range margins exhibit less genetic diversity and greater inter-population genetic differentiation compared to range cores. Since the Eastern Mediterranean shores are not a naturally occurring edge of the population this effect can only be seen in the Atlantic where *C. stellatus* does not spread further south to the Western Africa shore and even the Cape Verde *Chthamalus* was shown to be a different species (Tikochinski et al., 2020).

Yet another explanation may be the advantage of variability within the eastern basin populations in overcoming the higher temperature changes as well as other rapid condition changes and processes typical to this part of the Mediterranean, especially since the opening of the Suez Canal.

Our study presents a unique opportunity to study processes of population settlements in sessile animals, the influence of oceanographic conditions and processes including selection and genetic variation. In order to better understand and solidify some of our speculations, more studies of this range of geographic distribution, population size and genetic polymorphism are needed.

##  Supplemental Information

10.7717/peerj.11826/supp-1 Supplemental Information 1 Inventory collection numbers of samples of *Chthamalus stellatus.* Deposited in the Hebrew University of Jerusalem collection.

10.7717/peerj.11826/supp-2 Supplemental Information 2 Distances between the 14 populations according to the Genotype Based Method, the Haplotype Based Method and the SNP population based method

## Additional Information and Declarations

### DNA Deposition

The following information was supplied regarding the deposition of DNA sequences: The sequences used in our study are available at GenBank: MT296012–MT296247 and MT633576–MT633654 for EF1, MT296286–MT296518 and MT633655–MT633737 for NaKA.

### Data Availability

The following information was supplied regarding data availability: Raw measurements are available in the Supplemental File.

## References

[ref-1] Barnes H, Barnes M (1965). Egg size, nauplius size, and their variation with local, geographical, and specific factors in some common cirripedes. Journal of Animal Ecology.

[ref-2] Bhatnagar KM, Crisp DJ (1965). The salinity tolerance of nauplius larvae of cirripedes. Journal of Animal Ecology.

[ref-3] Burrows MT, Hawkins SJ, Southward JA (1992). A comparison of reproduction in co-occurring chthamalid barnacles, *Chthamalus stellatus* (Poli) and *Chthamalus montagui* Southward. Journal of Experimental Marine Biology and Ecology.

[ref-4] Burrows MT, Hawkins SJ, Southward AJ (1999). Larval development of the intertidal barnacles *Chthamalus stellatus* and *Chthamalus montagui*. Journal of the Marine Biological Association of the United Kingdom.

[ref-5] Carson HL (1955). The genetic characteristics of marginal populations of Drosophila. Cold Spring Harbor Symposia on Quantitative Biology.

[ref-6] Chan BKK, Chen H-N, Dando PR, Southward AJ, Southward EC (2016). Biodiversity and biogeography of Chthamalid barnacles from the North-Eastern Pacific (Crustacea Cirripedia). PLOS ONE.

[ref-7] Chen Y, Mao J, Senanan W, Wang W (2020). Identification of a large dataset of SNPs in *Larimichthys polyactis* using high-throughput 2b-RAD sequencing. Animal Genetics.

[ref-8] Crisp DJ, Southward AJ, Southward EC (1981). On the distribution of the intertidal barnacles *Chthamalus stellatus*, *Chthamalus montagui* and *Euraphia depressa*. Journal of the Marine Biological Association of the United Kingdom.

[ref-9] Ergon Å, Skøt L, Sæther VE, Rognli OA (2019). Allele frequency changes provide evidence for selection and identification of candidate loci for survival in red clover (*Trifolium pratense* L.). Frontiers in Plant Science.

[ref-10] Felsenstein J, Churchill GA (1996). A Hidden Markov Model approach to variation among sites in rate of evolution. Molecular Biology and Evolution.

[ref-11] Hamad N, Millot C, Taupier-Letage I (2006). The surface circulation in the eastern basin of the Mediterranean Sea. Scientia Marina.

[ref-12] Hsu KJ, Ryan WFB, Cita MB (1973). Late Miocene desiccation of the Mediterranean. Nature.

[ref-13] Johannesson K (1988). The paradox of Rockall: why is a brooding gastropod (*Littorina saxatilis*) more widespread than one having a planktonic larval dispersal stage (*L. littorea?*. Marine Biology.

[ref-14] Krijgsman W, Hilgen FJ, Raffi I, Sierro FJ, Wilson DS (1999). Chronology, causes and progression of the Messinian salinity crisis. Nature.

[ref-15] Longo GC, Lam L, Basnett B, Samhouri J, Hamilton S, Andrews K, Williams G, Goetz G, McClure M, Nichols KM (2020). Strong population differentiation in lingcod (*Ophiodon elongatus*) is driven by a small portion of the genome. Evolutionary Applications.

[ref-16] Mao L, Fang Y, Campbell M, Southerland WM (2017). Population differentiation in allele frequencies of obesity-associated SNPs. BMC Genomics.

[ref-17] Millot C, Taupier-Letage I, Saliot A (2005). Circulation in the Mediterranean Sea. Handbook of environmental chemistry.

[ref-18] Pannacciulli FG, Maltagliati F, De Guttry C, Achituv Y (2017). Phylogeography on the rocks: the contribution of current and historical factors in shaping the genetic structure of *Chthamalus montagui* (Crustacea, Cirripedia). PLOSONE.

[ref-19] Pannacciulli FG, Manetti G, Maltagliati F (2009). Genetic diversity in two barnacle species, *Chthamalus stellatus* and *Tesseropora atlantica* (Crustacea, Cirripedia), with different larval dispersal modes in the archipelago of the Azores. Marine Biology.

[ref-20] Pannacciulli FG, Bishop JDD, Hawkins SJ (1997). Genetic structure of populations of two species of *Chthamalus* (Crustacea: Cirripedia) in the north-east Atlantic and Mediterranean. Marine Biology.

[ref-21] Patarnello T, Volckaert FAMJ, Castilho R (2007). Pillars of Hercules: is the Atlantic–Mediterranean transition a phylogeographical break?. Molecular Ecology.

[ref-22] Pinardi N, Arneri E, Crise A, Ravaioli M, Zavatarelli M, Robinson AR, Brink KH (2006). The physical, sedimentary and ecological structure and variability of shelf areas in the Mediterranean Sea. The sea, ideas and observations on progress in the study of the Seas.

[ref-23] Poulain P-M, Bussani A, Gerin R, Jungwirth R, Mauri E, Menna M (2013). Mediterranean surface currents measured with drifters: From basin to subinertial scales. *Oceanography*.

[ref-24] Rozas J, Ferrer-Mata A, Sánchez-DelBarrio JC, Guirao-Rico S, Librado P, Ramos-Onsins SE, Sánchez-Gracia A (2017). DnaSP 6: DNA sequence polymorphism analysis of large datasets. Molecular Biology and Evolution.

[ref-25] Shemesh E, Huchon D, Simon-Blecher N, Achituv Y (2009). The distribution and molecular diversity of the Eastern Atlantic and Mediterranean chthamalids (Crustacea, Cirripedia). Zoologica Scripta.

[ref-26] Southward AJ (1976). On the taxonomic status and distribution of *Chthamalus stellatus* (Cirripedia) in the north-east Atlantic region: with a key to the common intertidal barnacles of Britain. Journal of the Marine Biological Association of the United Kingdom.

[ref-27] Stubbings HG (1967). The cirriped fauna of tropical West Africa. Bulletin of the British Museum (Natural History).

[ref-28] Tikochinski Y, Motro U, Simon-Blecher N, Achituv Y (2020). Molecular analysis reveals a cryptic species of *Chthamalus* in Cape Verde Islands. Zoological Journal of the Linnean Society.

[ref-29] Tikochinski Y, Russell B, Hyams Y, Motro U, Golani D (2016). Molecular analysis of the recently described lizardfish *Saurida lessepsianus* (Synodontidae) from the Red Sea and the Mediterranean, with remarks on its phylogeny and genetic bottleneck effect. Marine Biology Research.

[ref-30] Tsang LM, Wu TH, Shih HT, Williams GA, Chu KH, Chan KBK (2012). Genetic and morphological differentiation of the Indo-West Pacific intertidal barnacle *Chthamalus malayensis*. *Integr*. Comp. Biol..

[ref-31] Tsang LM, Chan BKK, Chatterjee T, Wu TH, Ng WC (2008). Population differentiation of the barnacle *Chthamalus malayensis*: postglacial colonization and recent connectivity across Pacific and Indian Oceans. Marine Ecology Progress Series.

[ref-32] Tsang LM, Chan BKK, Williams GA, Chu KH (2013). Who is moving where? Molecular evidence reveals pattern of range shift in the acorn barnacle *Hexechamaesipho pilsbryi* in Asia. Marine Ecology Progress Series.

[ref-33] UNEP/MAP (2012). State of the Mediterranean Marine and Coastal Environment, UNEP/MAP–Barcelona Convention, Athens, 2012.

[ref-34] Villamor A, Costantini F, Abbiati M (2014). Genetic structuring across marine biogeographic boundaries in rocky shore invertebrates. PLOS ONE.

[ref-35] Wares JP, Pansky MS, Pitombo F, Daglio LG, Achituv Y (2009). A shallow phylogeny of shallow barnacles (*Chthamalus*). PLOS ONE.

[ref-36] Weist P, Damerau MSchadeFM, Barth JMI, Dierking J, André C, Petereit C, Reusch T, Jentoft S, Hanel R, Krumme U (2019). Assessing SNP-markers to study population mixing and ecological adaptation in Baltic cod. PLOS ONE.

[ref-37] Winston JF (2012). Dispersal in marine organisms without a pelagic larval phase. Integr. Comp. Biol..

[ref-38] Wu TH, Tsang LM, Chan BKK, Chu KH (2015). Cryptic diversity and phylogeography of the island-associated barnacle *Chthamalus moro* in Asia. Marine Ecology.

