# Supplementary material for: A star is torn—molecular analysis divides the Mediterranean population of Poli’s stellate barnacle, Chthamalus stellatus (Cirripedia, Chtamalidae)
Source: PeerJ. 2021 Jul 21;9:e11826. doi: 10.7717/peerj.11826 (PMC8308608; doi:10.7717/peerj.11826)
Supplement: Supplemental Information 1 — Deposited in the Hebrew University of Jerusalem collection. [file peerj-09-11826-s001.docx]

| Locality |  |  | Coordinates | HUJI Zoo. Collec. # |
| --- | --- | --- | --- | --- |
| Bodrum | Aegean Sea, | Turkey | [37°02′N 27°26′E](https://geohack.toolforge.org/geohack.php?pagename=Bodrum&params=37_02_00_N_27_26_00_E_type:city_region:TR-48) | HUJINVCRUSTCIR327 |
| Dubrovnik | mid Adriatic Sea | Croatia | [42°38′N 18°06′E](https://geohack.toolforge.org/geohack.php?pagename=Dubrovnik&params=42_38_25_N_18_06_30_E_region:HR_type:city(42615)) | HUJINVCRUSTCIR364 |
| Fažana | north Adriatic Sea | Croatia | [44°56′N 13°48′E](https://geohack.toolforge.org/geohack.php?pagename=Fa%C5%BEana&params=44_56_N_13_48_E_region:HR_type:city(3569)) | HUJINVCRUSTCIR325 |
| Larnaca |  | Cyprus | [34°55′N 33°38′E](https://geohack.toolforge.org/geohack.php?pagename=Larnaca&params=34_55_N_33_38_E_region:CY_type:city) | HUJINVCRUSTCIR329 |
| Rethymno | Crete | Greece | [35°22′N 24°28′E](https://geohack.toolforge.org/geohack.php?pagename=Rethymno&params=35_22_N_24_28_E_type:city_region:GR-M) | HUJINVCRUSTCIR330 |
| Bastia | Corsica | France | [42°42′N 9°27′E](https://geohack.toolforge.org/geohack.php?pagename=Bastia&params=42.7008_N_9.4503_E_type:city(45715)_region:FR-COR) | HUJINVCRUSTCIR331 |
| Málaga | North Alboran Sea | Spain | 3[6°43′N 4°25′W](https://tools.wmflabs.org/geohack/geohack.php?pagename=%D7%9E%D7%90%D7%9C%D7%92%D7%94&language=he&params=36.716666666667_N_-4.4166666666667_E_type:city) | HUJINVCRUSTCIR332 |
| Melilla | South Alboran Sea | Spain | [35°18′N 2°57′W](https://geohack.toolforge.org/geohack.php?pagename=Melilla&params=35_18_N_2_57_W_region:ES-ML_type:city) | HUJINVCRUSTCIR333 |
| Birżebbuġa |  | Malta | [35°49′N 14°31′E](https://geohack.toolforge.org/geohack.php?pagename=Bir%C5%BCebbu%C4%A1a&params=35_49_32_N_14_31_41_E_type:city_region:MT) | HUJINVCRUSTCIR334 |
| Bizerte |  | Tunisia | [37°16′N 9°51′E](https://geohack.toolforge.org/geohack.php?pagename=Bizerte&params=37_16_40_N_9_51_50_E_region:TN_type:city) | HUJINVCRUSTCIR335 |
| Pantelleria | Strait of Sicily | Italy | [36°47′N 11°59′E](https://geohack.toolforge.org/geohack.php?pagename=Pantelleria&params=36_47_15_N_11_59_33_E_region:IT-TP_type:isle) | HUJINVCRUSTCIR348 |
| Biarritz | Bay of Biscay | France | 44′°50′N 00°34′E | HUJINVCRUSTCIR337 |
| Gran Canaria, | Canary Islands | Spain | [27°58′N 15°36′W](https://tools.wmflabs.org/geohack/geohack.php?pagename=%D7%91%D7%99%D7%90%D7%A8%D7%99%D7%A5&language=he&params=44_50_19_N_00_34_42_W_type:city) | HUJINVCRUSTCIR338 |
| Funchal | Madeira | Portugal | [32°39′N 16°55′W](https://geohack.toolforge.org/geohack.php?pagename=Funchal&params=32_39_N_16_55_W_region:PT_type:adm1st_dim:100000) | HUJINVCRUSTCIR339 |
